# Supplementary material for: Incubation Period and Other Epidemiological Characteristics of 2019 Novel Coronavirus Infections with Right Truncation: A Statistical Analysis of Publicly Available Case Data
Source: J Clin Med. 2020 Feb 17;9(2):538. doi: 10.3390/jcm9020538 (PMC7074197; doi:10.3390/jcm9020538)
Supplement: Supplementary file 1 [file jcm-09-00538-s001.zip › linton_supp_textS1_8Feb2020.docx]

Supplementary Text S1

1. Estimation of the time interval distribution using doubly interval-censored likelihood

Here we consider the fit of the incubation period as the time interval between the windows of exposure *“e”* and symptom onset *“s”* (written in lower or upper case). This approach can be applied to other time intervals, such as the interval between illness onset and death, illness onset and hospitalization, etc. The doubly interval-censored likelihood holds the following form:

|  | $L\left( \boldsymbol{\Theta} \right\vert\boldsymbol{D})=\prod_{i} \int_{E_{L,i}}^{E_{R,i}} \int_{S_{L,i}}^{S_{R,i}} g\left( e \right)f'(s-e,e)dsde,$ | (S1) |
| --- | --- | --- |

where *L* refers to the left-hand (lower) value of a window of exposure or symptom onset, and *R* is the right-hand (upper) value; *i* is the index over all available data record of pairs $\left( S_{L,i},S_{R,i} \right)$ and $\left( E_{L,i},E_{R,i} \right)$ with the assumption that at least $S_{R_{i}}>E_{L_{i}}$. A previously published work by Reich and colleagues [1] considered the same problem but under the condition of a uniformly distributed exposure distribution and $f'\left( s-e,e \right)\equiv f(s-e)$. The results of Reich and colleagues can be altered to include a more general form of the function under the integral. Here, we describe how this can be done.

The main difficulty in calculating the likelihood (S1) is addressing the double integral over the rectangular area $\left[ S_{L,i},S_{R,i} \right]\times\left[ E_{L,i},E_{R,i} \right]$:

|  | $\mathcal{I}_{i}=\int_{E_{L,i}}^{E_{R,i}} \int_{S_{L,i}}^{S_{R,i}} g\left( e \right)f'(s-e,e)dsde.$ | (S2) |
| --- | --- | --- |

We notice that the integral $\mathcal{I}_{i}$ can be transformed to the repeated integral after changing the variable $s^{'}=s-e$, and subsequently calculated using Figure 1.

Figure 1A shows the situation when $S_{L,i}>E_{R_{i}}$. In this case:

|  | $\mathcal{I}_{i}=\int_{E_{L,i}}^{E_{R,i}} de g\left( e \right)\int_{S_{L,i}-e}^{S_{R,i}-e} f^{'}\left( s',e \right)ds'=\int_{E_{L,i}}^{E_{R,i}} g\left( e \right)\left( F^{'}\left( S_{R,i}-e,e \right)-F^{'}\left( S_{L,i}-e,e \right) \right)de,$ | (S3) |
| --- | --- | --- |

where $F'$ is a cumulative distribution function (CDF) of $f'$.

Figure 1B shows the situation when $E_{R_{i}}>S_{L,i}>E_{L,i}.$. In this case:

|  | $\mathcal{I}_{i}=\int_{E_{L,i}}^{S_{L,i}} g\left( e \right)\left( F^{'}\left( S_{R,i}-e,e \right)-F^{'}\left( S_{L,i}-e,e \right) \right)de+\int_{S_{L,i}}^{E_{R,i}} g\left( e \right)F^{'}\left( S_{R,i}-e,e \right) de.$ | (S4) |
| --- | --- | --- |

Lastly, Figure 1C shows the situation when $E_{L_{i}}>S_{L,i}.$ In this instance:

|  | $\mathcal{I}_{i}=\int_{E_{L,i}}^{E_{R,i}} g\left( e \right)F^{'}\left( S_{R,i}-e,e \right) de.$ | (S5) |
| --- | --- | --- |

We note that the doubly interval-censored likelihood with truncation admits the function $f'$ to be separable on its arguments: $f^{'}\left( s-e,e \right)=f\left( s-e \right)h\left( e \right)$. This means that the CDF: $F^{'}\left( s-e,e \right)=F\left( s-e \right)h\left( e \right)$ contains the CDF of the function $f(s-e)$ and can be analytically derived for all three gamma, lognormal, and Weibull distributions considered in the main text.

Our numerical simulations confirmed that calculation of the likelihood using the formulas (S3)–(S5) lead to results that are identical to those produced using the R package coarseDataTools developed by Reich and colleagues [2].


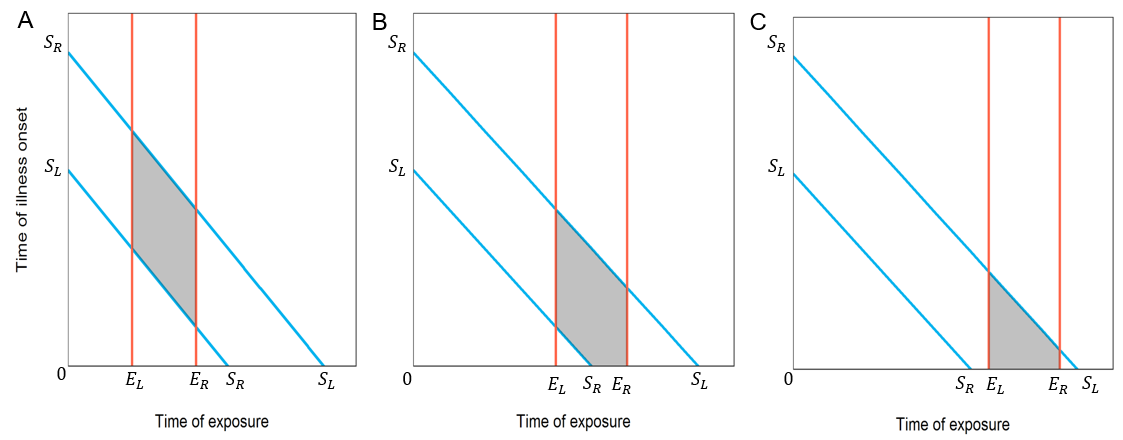


**Figure S1. Illustration of the time interval distribution using doubly interval-censored likelihood.** The figure depicts how doubly interval-censored likelihood was calculated when A) $S_{L,i}>E_{R_{i}}$, B) $E_{R_{i}}>S_{L,i}>E_{L,i}$ and C) $E_{L_{i}}>S_{L,i}$. The blue lines and red lines indicate the lower or upper bounds of illness onset and exposure, respectively. The gray shaded area represents the integral in each situation.

# **B. Estimation of the time interval distributions using Bayesian framework.**

Here, we estimate parameters of time interval distributions for non-truncated likelihood using Markov chain Monte Carlo (MCMC) simulations. We used Stan packages with implementation of No U-Turn Sampler (NUTS). Specifically, each distribution is specified by the following prior distributions:

- The lognormal distribution is defined by the logarithms of the mean and the logarithm of the standard deviation. Both follow the standard normal distribution (with mean set at zero, and standard deviation equal to one).
- The Weibull distribution is defined by the logarithm of the mean and the logarithm of the shape parameter. Both follow the standard normal distribution.
- The gamma distribution is defined by the shape and inverse scale parameter. The former follows the normal distribution with the mean three and standard deviation five and is constrained to positive values. The latter follows a Cauchy distribution with location parameter set to zero and scale parameter equal to 5.0.

We followed general recommendations of the Stan developer community for the choice of prior distributions [3].

Additionally, we specified the priors for the times of exposure and illness onset (if necessary, relative to the other time interval distributions) for each case $i$:

$$e_{i}=E_{L,i}+\left( E_{R,i}-E_{L,i} \right)\tilde{e}_{i}, s_{i}=\hat{S}_{L,i}+\left( S_{R,i}-\hat{S}_{L,i} \right)\tilde{s}_{i},$$

where: $\hat{S}_{L,i}=e_{i}$ if $e_{i}>S_{L,i},$ and $\hat{S}_{L,i}=S_{L,i},$ otherwise, and $\tilde{e}_{i}$ and $\tilde{s}_{i}$ are a pair of standardized variables:

$$\tilde{e}_{i} \sim\mathrm{normal}\left( \mathrm{mean}=0.5, SD=0.5 \right), \tilde{s}_{i} \sim\mathrm{normal}\left( \mathrm{mean}=0.5,\mathrm{SD}=0.5 \right),$$

on the interval between zero and one.

The non-truncated loglikelihood written in terms of the Stan language was defined as:

$$\log L(\boldsymbol{\Theta})=\sum_{i} distibution\_lpdf\left( s_{i}-e_{i} | \boldsymbol{\Theta} \right),$$

where $distibution\_lpdf$ is either the function $lognormal\_lpdf$, $weibull\_lpdf$, or $gamma\_lpdf$; “lpdf” is shorthand for the logarithm of the probability distribution function.

We also used a slight variation of this model for the incubation period as to include censored likelihood to account for unknown lower bound of the exposure period.

In each case, we ran four chains of MCMC simulations consisting of 5,000 iterations for the simulation phase and 10,000 used for the tuning phase. The convergence of the obtained traces was verified visually, and we also explored the r-hat values and effective sample size. For example, Figure 2 shows the traces obtained for the fit of the incubation period to a lognormal distribution, which was the best-fit model as seen in Table 1 of the main text.


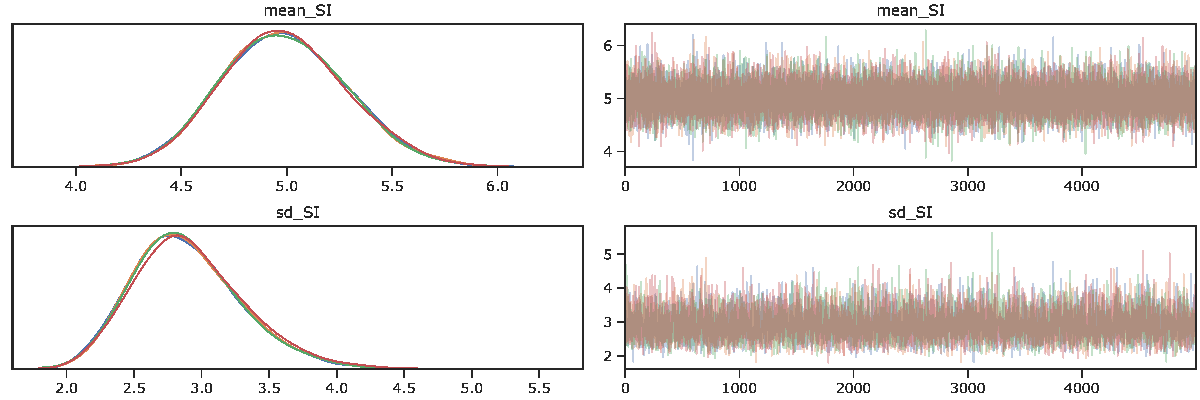


**Figure S2. The density plots and traces for the mean and standard deviation of the lognormal distribution when fitted to the dataset of incubation periods including Wuhan residents (second column of Table 1).**

1. Data cleaning rules implemented for the various time intervals

Incubation period:

For incubation periods of non-Wuhan residents (exposure type !=“Lives/works/studies in Wuhan”):

- Only include cases with travel to Wuhan, contact with Wuhan residents, or contact with a case.

- Must have a left exposure date (EL).

- One of onset, date first sought healthcare, or date hospitalized/isolated must be larger than left exposure date.

- For calculating ER and SL, must have right exposure date or onset date.

- For calculating SR, must have onset date or date first sought healthcare or date hospitalized/isolated or date of report.

- If left exposure (EL) is the same as the right exposure (ER)—which was the case for some people with day trips to Wuhan or contact with cases—we set ER to EL + 1 to provide a “day” of exposure.

- If right exposure date was not available or was greater than date of onset, ER was set to date of onset.

- Left onset (SL) is the date of onset (if available), otherwise set to the day after ER.

- Right onset (SR) is the day after date of onset if available, otherwise set to the date first sought healthcare, date hospitalized/isolated, or date reported as case, in that order.

For incubation period including Wuhan residents, the only difference in methods is that we do not require left exposure date for Wuhan residents. If left exposure date is NA, they must have exposure type “Lives-works-studies in Wuhan” or are excluded. Then, if the left exposure date is missing and they are a Wuhan resident, E_L is set to 1 December 2019, which was the first date of onset for a reported case from Wuhan [4].

Onset to hospitalization:

- Calculated separately for deceased cases (almost all Hubei residents) and cases diagnosed outside of Hubei Province:

- Must have date of onset.

- Must have date of hospitalization or date of report (death for death dataset).

- Left onset (EL) is the date of onset.

- Right onset (ER) is the date of onset + 1.

- Left hospitalization/isolation (SL) is date of hospitalization, if available, otherwise date first sought healthcare, otherwise date of onset + 1.

- Right hospitalization/isolation (SR) is date of hospitalization + 1 if available, otherwise date of report (for export cases) or death (for deceased cases).

Onset to death:

- Must have date of onset.

- Must have date of death.

- Left onset (EL) is the date of onset.

- Right onset (ER) is the date of onset + 1.

- If time of death is known, left date of death (SL) is the date of death + time of death - 1 hour, otherwise date of death - 1 day.

- If time of death is known, right date of death (SR) is the date of death + time of death, otherwise date of death.

Hospitalization to death:

- Must have date of hospitalization or onset.

- Must have date of death.

- Left hospitalization/isolation (SL) is date of hospitalization, if available, otherwise date first sought healthcare, otherwise date of onset + 1.

- Right hospitalization/isolation (SR) is date of hospitalization + 1 if available, otherwise date of death - 1.

- If time of death is known, left date of death (SL) is the date of death + time of death - 1 hour, otherwise date of death - 1 day.

- If time of death is known, right date of death (SR) is the date of death + time of death, otherwise date of death.

# **References**

1. Reich NG.; Lessler J.; Cummings D.A.T.; Brookmeyer R. Estimating incubation period distributions with coarse data. Stat Med. 2009, 28:2769–84.
2. Reich NG.; Lessler J.; Azman A. coarseDataTools: A collection of functions to help with analysis of coarsely observed data. R package version 0.6-5, <https://cran.r-project.org/package=coarseDataTools>. 2019.
3. Stan developer team. Prior choice recommendations. <https://github.com/stan-dev/stan/wiki/Prior-Choice-Recommendations>. Assessed 6 February 2020.
4. Li, Q., Guan, X., Wu, P., Wang, X., Zhou, L., Tong, Y., … Feng, Z. Early transmission dynamics in Wuhan, China, of novel coronavirus–infected pneumonia. New England Journal of Medicine. 2020, 1–9. https://doi.org/10.1056/NEJMoa2001316.
